# ﻿*Ictalurusnazas* sp. nov., a new species of North American catfish (Siluriformes, Ictaluridae) from Mexico

**DOI:** 10.3897/zookeys.1248.151641

**Published:** 2025-08-04

**Authors:** Edith Avila-Treviño, Gabriel Fernando Cardoza-Martínez, Fernando Alonzo-Rojo, Rodolfo Pérez-Rodríguez

**Affiliations:** 1 Facultad de Ciencias Biológicas, Universidad Juárez del Estado de Durango, Av. Universidad S/N Fracc. Filadelfia, Gómez Palacio, 35010 Durango, Mexico Universidad Juárez del Estado de Durango Gómez Palacio Mexico; 2 Laboratorio de Biología Acuática, Facultad de Biología, UMSNH, 58000 Morelia, Michoacán, Mexico Laboratorio de Biología Acuática, Facultad de Biología Morelia Mexico

**Keywords:** *Ictaluruspricei*, identification key, morphological comparison, Nazas basin, *punctatus* group, Siluriformes, taxonomy

## Abstract

Catfishes of the genus *Ictalurus* (Ictaluridae) range geographically from southern Canada to northern Guatemala and Belize. The systematics of this genus remain unresolved, with recent studies suggesting the presence of cryptic diversity. This is the case for a potentially undescribed catfish species distributed in the Nazas River basin, which has been recorded to date as a population of *Ictaluruspricei*. Recent phylogenetic studies suggest that it represents an independently evolving lineage that is distinct from *I.pricei*. This study aimed to determine the diagnostic characters that differentiate the Nazas River basin lineage and to describe this lineage as a new taxon. Morphological comparisons were made using meristic and morphometric characters. The study identified useful meristic and morphometric characters for diagnosing *Ictalurusnazas***sp. nov.**, which differentiate it from *I.pricei*.

## ﻿Introduction

Ictaluridae currently comprises 50 catfish species distributed across seven genera (Burr et al. 2020) and, together with Leuciscidae (191 species/33 genera; Schönhuth et al. 2018), Percidae (190 species/7 genera; Carlson and Wainwright 2010; Stepien and Haponski 2015), and Catostomidae (75 species/12 genera; Harris et al. 2014), it is considered one of the most species-rich families of freshwater fishes in North America. Since Taylor (1954) proposed the name Ictaluridae for North American catfishes and bullheads, this familial classification has undergone historical changes due to the instability caused by the addition of new information (morphological and molecular data), as well as its lack of robustness (resulting from substantial modifications with the inclusion of new entities). This is evidenced by the incorporation of new characters and entities, populations and/or taxa. Such is the case of the genus *Ameiurus* Rafinesque, 1820b, which was synonymized with *Ictalurus* Rafinesque, 1820b by Taylor (1954) and Taylor and Lundberg (1969) and then reinstated to the genus level by Lundberg (1992), who incorporated a phylogenetic approach and additional morphological characters. Subsequent phylogenies based on molecular (Hardman and Page 2003; Hardman and Hardman 2008; Arce-H. et al. 2016; Pérez-Rodríguez et al. 2023; Janzen et al. 2023) and morphological (Arce-H. et al. 2016) data corroborated this classification within Ictaluridae. Recently, the increased volume of molecular data and the application of several new phylogenetic approaches, incorporating mitochondrial and nuclear markers to provide a more detailed evolutionary framework, have revealed limitations of the classification based solely on morphological traits and outdated methodological approaches. For example, ictalurid cave species exhibit morphological similarities due to adaptations to the extreme conditions of their habitats (e.g., absence of light), which have historically led researchers to group them into a single clade. However, the polytypic genus *Prietella* Carranza, 1954 was later found to be polyphyletic, indicating that *Prietellalundbergi* Walsh & Gilbert, 1995 should be removed from this genus (Wilcox et al. 2004; Janzen et al. 2023).

Within the genus *Ictalurus*, questions have arisen from observations of comparative materials for taxonomic identification in ichthyofaunal catalogs, new records, or expert personal assessments regarding the validation of certain species, while also revealing the presence of undescribed species (Lundberg 1992). Several of these findings have been corroborated by recent phylogenetic hypotheses (Janzen et al. 2023; Pérez-Rodríguez et al. 2023). One such undescribed species is *Ictalurus* sp. from the Nazas River basin, which was originally recognized as a population of *Ictaluruspricei* (Rutter, 1896) (Meek 1904; Álvarez del Villar 1970), a widely distributed species with several geographically isolated populations that have recently been found to be molecularly well-differentiated (Castañeda-Rivera et al. 2014; Ballesteros-Córdova et al. 2015; Janzen et al. 2023; Pérez-Rodríguez et al. 2023). Among the undescribed populations of *I.pricei*, those of the Nazas River basin are notable since they are more closely related to *Ictalurusdugesii* (Bean, 1880) and *Ictaluruslupus* (Girard, 1858), within the *punctatus* group (Janzen et al. 2023; Pérez-Rodríguez et al. 2023). Furthermore, in contrast to other *I.pricei* populations, the origins of which date back to the Pleistocene, the cladogenesis of *Ictalurus* sp. from the Nazas River basin occurred during the early Pliocene (Janzen et al. 2023; Pérez-Rodríguez et al. 2023). *Ictalurus* sp. from the Nazas River has even been considered an undescribed species by some authors (Miller 1986; Smith and Miller 1986). However, no taxonomic revision or morphological comparison has been conducted to provide useful characters for the diagnosis of *Ictalurus* sp. from the Nazas River basin as a distinct taxon.

The aim of this study was therefore to perform a morphological comparison between *Ictalurus* sp. and *I.pricei*, the nominal species that has historically included *Ictalurus* sp. from the Nazas River basin, and to identify useful morphological characters to diagnose this undescribed species from other members of the genus *Ictalurus* and formally describe it as a new taxon.

## ﻿Materials and methods

**Figure 1.**
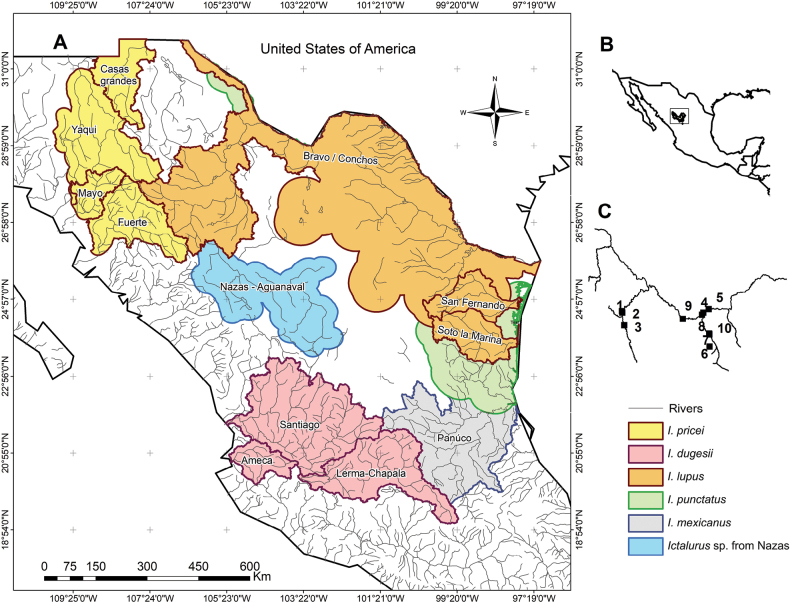
Distributions of recognized species of the *Ictaluruspunctatus* group in Mexico. A. River basins in northern and central Mexico inhabited by species of the *punctatus* group (sensu Pérez-Rodríguez et al. 2023); B. Map of northern and central Mexico showing the distribution of *Ictalurusnazas* sp. nov.; C. Nazas River basin with sampling sites of the specimens used in this study. The numbers correspond to the locations listed in Table 1.

Specimens of *Ictalurus* sp. were collected throughout the Nazas River basin (Fig. 1, Table 1). All specimens were fixed in 5% formalin, preserved in 70% ethanol, and deposited in the fish collection of the Universidad Juárez del Estado de Durango (FCB-UJED). Specimens deposited in “Colección de Peces de la Universidad Michoacana (CPUM), Facultad de Biología, Universidad Michoacana de San Nicolás de Hidalgo” and “Colección Nacional de Peces (CNP), Instituto de Biología, Universidad Nacional Autónoma de México” were also analyzed.

**Table 1.** Locality data and catalog information of specimens used for morphological comparisons. Locality numbers correspond to the sites within the Nazas river basin shown in Fig. 1C.

	Locality	Drainage	Coordinates	Catalog
1	1.12 km north of El Olote, Santiago Papasquiaro, Durango	Ramos River	25°14'13.2"N, -105°27'0.4"W	CPUM-2654
2	El Olote, Santiago Papasquiaro, Durango	Ramos River	25°13'17.04"N, -105°26'37.79"W	CPUM-7266
3	River east of Papasquiaro, Durango	Ramos River	25°2'26.12"N, -105°24'54.67"W	CPUM-1642
4	Nazas River, main body, Nazas, Durango	Nazas River	25°12'57"N, -104°10'25"W	CNPE-IBUNAM-17795
5	Dolores Hidalgo, Nazas, Durango	Nazas River	25°15'59"N, -104°05'24"W	CNPE-IBUNAM-17779
6	Balneario “Belem” Peñón Blanco, Durango	Nazas River	24°44'36"N, -104°04'36"W	CPUJED-0018
7	El Ranchito, Peñón Blanco, Durango	Nazas River	24°59'19"N, -104°19'39"W	CPUJED-0001
8	Arroyo Covadonga, Nazas, Durango	Nazas River	25°11'31"N, -104°11'41"W	CPUJED-0002
9	Amoles, Nazas, Durango	Nazas River	25°07'47"N, -104°29'47"W	CPUJED-0003
10	Nuevo Covadonga, Peñón Blanco, Durango	Nazas River	24°54'35"N, -104°04'47 W	CPUJED-0004
	Aros River, to the south of Natora, Son., Sahuipa, Sonora, Mexico	Yaqui River	29°0'59"N, -108°45'29"W	CNPE-IBUNAM-5712
	Bavispe River to 8.85 km east of Col. Morelos, Bavispe, Sonora, Mexico	Yaqui River	30°48'49.6"N, -109°7'45.3"W	UMSNH-2931
	Moctezuma River south of Caserio de Terapa, Moctezuma, Sonora, Mexico	Yaqui River	29°40'41.7"N, -109°39'24.6"W	UMSNH-7763
	Bavispe River 7.2 km northeast of the village de Huachinera, Bacerac, Sonora, Mexico	Yaqui River	30°16'14.2"N, -108°55'26.3"W	UMSNH-2221
	Guerachi River to 27 km southeast of Guachochi city, Guadalupe y Calvo, Chihuahua	Fuerte River	26°40'41.6"N, -107°19'24.6"W	UMSNH-2642
	Guerachi River 27 km southeast of Guachochi city, Guadalupe y Calvo, Chihuahua, Mexico	Fuerte River	26°40'41.6"N, -107°19'24.6"W	UMSNH-2534
	Guerachi River 27 km southeast of Guachochi city, Guadalupe y Calvo, Chihuahua, Mexico	Fuerte River	26°40'41.6"N, -107°19'24.6"W	UMSNH-7764
	El Sauzal, Urique, Chihuahua, Mexico	Fuerte River	27°12'09"N, -107°54'49"W	CPUJED-0008
	San Isidro Stream, Rosario, Sinaloa, Mexico	Fuerte River	–	CNPE-IBUNAM-20776

Meristic counts and linear measurements were taken from the left side of 24 specimens of *Ictalurus* sp. from the Nazas River basin (hereafter *Ictalurus* sp. from Nazas) and 36 specimens of *I.pricei* with a standard length exceeding 100 mm (Table 1), following Hubbs and Lagler (1958). Meristic counts included rays and spines in the dorsal (D), caudal (C), anal (A), pectoral (P), and pelvic (PV) fins. Moreover, three traits were considered derived from the characterization of the pectoral spine, as described by Arce-H. et al. (2016): the number and development of dentations, and their distribution along the posterior face of the pectoral spine. However, only the number of dentations was included in the analysis of meristic counts since it is the only one of these three traits that is discrete.

The linear measurements comprised the standard length (SL), head length (HL), caudal peduncle length (CPL), minimum (minH) and maximum (maxH) height, eye diameter (ED), interorbital distance (IO), anal fin base length (AFL), pectoral fin length (PFL), pelvic fin length (PvFL), body width (BW), and lower jaw length (LJL). All measurements were taken using digital calipers and recorded in mm. The data were then used to calculate the following proportions: head length to standard length, (SL/HL), interorbital distance to head length (HL/ID), eye diameter to head length (HL/ED), lower jaw length to head length (HL/LJL), lower jaw length to standard length (SL/LJL), caudal peduncle length to standard length (SL/CPL), maximum height to standard length (SL/MaxH), minimum height to standard length (SL/MinH), anal fin base length to standard length (SL/AFL), pectoral fin length to standard length (SL/PFL), pelvic fin length to standard length (SL/PvFL), and body width to standard length (SL/BW).

Multivariate and univariate approaches were employed to analyze both meristic and linear trait data to determine whether *Ictalurus* sp. from Nazas and *I.pricei* are morphologically disparate. A principal component analysis (PCA) was performed using the prcomp function built into R, based on correlation matrices. To determine significant differences in the proportions of linear measurements between *Ictalurus* sp. from Nazas and *I.pricei*, a Student’s t-test was performed using the t-test function built into R. All analyses were conducted in R v. 4.2.1 (R Core Team 2022).

To depict the independent evolutionary history and demonstrate, under a phylogenetic criterion (monophyly), the differentiation level exhibited by *Ictalurus* sp. from Nazas in relation to the hypothesized species of the genus (sensu Pérez-Rodríguez et al. 2023), particularly its relationship with *I.pricei*, we conducted a phylogenetic analysis of the genus *Ictalurus*. Phylogenetic analyses were based on the mitochondrial cytochrome *b* gene (*Cytb*) and the nuclear recombination-activation gen 1 (*RAG1*). All gene sequences were retrieved from GenBank and corresponded to specimens deposited in the Auburn University Museum Fish Collection (AUM), the Academy of Natural Sciences Ichthyology Collection (ANSP), the Ichthyology Collection of the Royal Ontario Museum (ROM), the Mississippi Museum of Natural Science (MMNS), the Florida Museum of Natural History (UF FLMNH), and the fish collection of the Universidad Michoacana de San Nicolás de Hidalgo (UMSNH). This highlights the fact that several sequences for both loci belong to voucher specimens of *Ictalurus* sp. from Nazas and *I.pricei* (Table 2).

**Table 2.** Geographic distribution range, voucher specimens, gene, and GenBank accession number of the analyzed sequences.

Taxa	Locality	Voucher tissue	Cytb	RAG1
Ictalurusdugesii	Angulo River at Sabino town; middle Lerma basin, Michoacan, Mexico	UMSNH 6182	ON008875	ON008735
		UMSNH 6224	ON008876	ON008736
Ictalurusfurcatus (Valenciennes, 1840)	Grijalva-Usumacinta River basin, Mexico	UMSNH 3232	OQ559162	OQ567222
	Apalachicola River, Florida, USA	UF 238203	OQ559161	OQ567221
Ictaluruslupus	San Juan-Bravo, Nuevo Leon, Mexico	UMSNH 57921	OQ559164	OQ567224
	Conchos alto, Durango, Mexico	UMSNH 41562	OQ559163	OQ567223
Ictalurusnazas sp. nov.	Nazas, Durango, Mexico	UMSNH 45533	OQ559173	OQ567234
		UMSNH 40907	OQ559172	OQ567233
	Ramos River, 1.4 km north of Ramos town; Nazas basin Durango, Mexico	UMSNH 1796	ON008915	ON008752
		UMSNH 1800	ON008916	ON008753
		UMSNH 1805	ON008917	ON008754
	El Olote town, Santiago Papasquiaro; Nazas basin, Durango, Mexico	UMSNH 5987	ON008918	ON008755
		UMSNH 6819	ON008919	ON008756
Ictaluruspricei	Bavispe River, 7.2 km northeast of Huachinera town; Yaqui basin, Chihuahua, Mexico	UMSNH 8002	ON008846	ON008709
	Bavispe River, east of San Miguelito town; Yaqui basin Chihuahua, Mexico	UMSNH 8011	ON008848	ON008710
	Guerachi River, 27 km southwest of Guachochi; Fuerte basin, Chihuahua, Mexico	UMSNH 7058	ON008849	ON008711
		UMSNH 7059	ON008850	ON008712
		UMSNH 7060	ON008851	ON008713
		UMSNH 7064	ON008852	ON008714
		UMSNH 7073	ON008853	ON008715
		UMSNH 7124	ON008854	ON008716
Ictaluruspunctatus (Rafinesque, 1818)	Bolanos-Santiago River basin, Mexico	UMSNH 5722	OQ559171	OQ567232
	Lake St Clair, Ontario, Canada	ROM 86705	OQ559170	OQ567231
Ictalurusmexicanus (Meek, 1904)	Gallinas River al Jabali town, San luis Potosi, Mexico	MNCN 2872	ON008921	ON008758
		MNCN 2873	ON008922	ON008759

The obtained sequences were aligned using MEGA X (Kumar et al. 2018) and subsequently checked and manually corrected. The substitution models that best fitted the mitochondrial and nuclear datasets were determined using the program JModelTest 2 (Darriba et al. 2012), based on the Bayesian Information Criterion (BIC). Phylogenetic reconstruction analyses were conducted for the *Cytb* and *RAG1* gene matrices using both the Bayesian inference and maximum likelihood methods. These analyses were performed with MrBayes v. 3.2 (Ronquist et al. 2012) and RAxML v. 8.2.12 (Stamatakis 2006), as implemented in CIPRES (Miller et al. 2010). Moreover, *Bagrusubangensis* Boulenger, 1902 *Cranoglanishenrici* (Vaillant, 1893), *Noturusgladiator* Thomas & Burr, 2004, *Pylodictisolivaris* (Rafinesque, 1818), and *Ameiurusmelas* (Rafinesque, 1820a) were used as outgroups (Suppl. material 1: table S1) for both analyses. Finally, uncorrected (*p*) genetic distances between species of the *Ictalurus* were calculated for both loci using MEGA X (Kumar et al. 2018).

## ﻿Results

**Figure 2.**
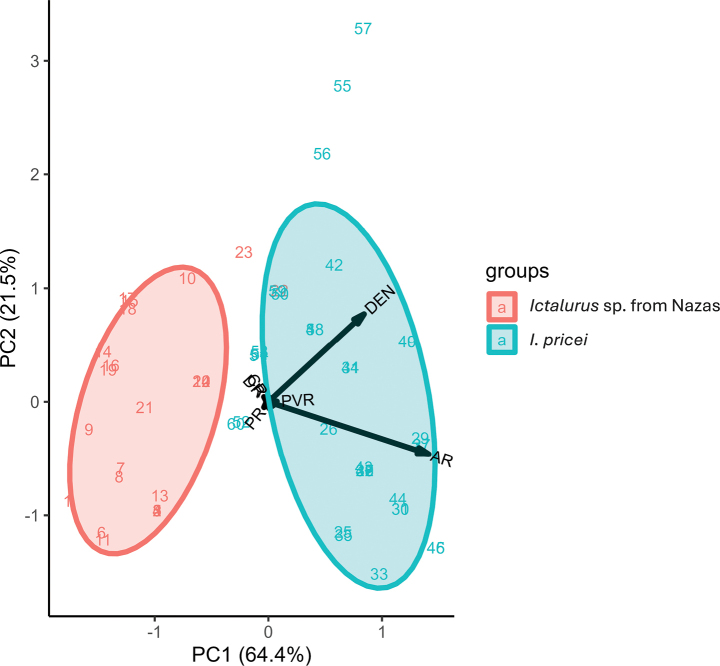
Principal Components Analysis of the meristic characters used for species discrimination between *Ictalurus* sp. from Nazas (*N* = 24) and *I.pricei* (*N* = 36).

Principal component analysis using meristic traits revealed two distinct groups corresponding to *Ictalurus* sp. from Nazas and *I.pricei*, when plotting the first and second components. The first and second components explained 64.4% and 21.5% of the explained variance, respectively (Fig. 2, Table 3). The characters with the greatest loads, mainly in the first component (C1), were the anal fin rays and the number of posterior dentations on the pectoral spine (Table 3). *Ictalurus* sp. from Nazas presented fewer anal fin rays (16–19) compared to *I.pricei* (19–24). Similarly, the number of posterior dentations on the pectoral spine was lower in *Ictalurus* sp. from Nazas (5–10) than in *I.pricei* (8–14) (Table 4). The remaining traits (development and distribution of dentations) of the pectoral spine serve to further differentiate the two species. The development of the dentations was weaker in *Ictalurus* sp. from Nazas compared to that of *I.pricei* (Fig. 6), and the distribution of the dentations in *Ictalurus* sp. from Nazas was concentrated towards the apical region of the pectoral spine whereas in *I.pricei*, these dentations extended from the apical region to beyond the median part of the pectoral spine (Fig. 6).

**Table 3.** Eigenvalues and eigenvectors for the first two principal components (PC1 and PC2) obtained from six meristic characters used for the discrimination of *Ictalurus* sp. from Nazas (*N* = 24) and *I.pricei* (*N* = 36).

	PC1	PC2
Eigenvalues	7.16	2.39
Percentage of variance explained	64.44	21.51
Eigenvectors		
Dorsal fin rays	-0.0245	0.0468
Caudal fin rays	-0.0204	0.0407
Anal fin rays	0.8716	-0.4852
Pectoral fin rays	-0.0226	-0.0481
Pelvic fin rays	0.0451	0.0033
Dentations	0.4865	0.8708

**Table 4.** Frequencies of rays in the dorsal, caudal, anal, pectoral, and pelvic fins and number of dentations on the pectoral spine.

	Dorsal rays							Caudal rays								Anal rays																			
	5	6			7	9		15		16		17			18		19		16		17		18		19		20		21		22		23		24
Ictalurus sp. from Nazas	2	16			5	1				2		16			1		5		8		6		9		1										
I.pricei	1	31			4			1		2		25			6		2								10		4		3		10		7		2
	Pectoral rays									Pelvic rays							Dentations																		
	6		7	8			9		10		6		7	8		9		5		6		7	8	9		10		11		12		13		14	
Ictalurus sp. from Nazas	1		4	11			7		1		1		5	17		1		3		8		4	6	1		2									
I.pricei	1		3	30			2						1	34		1							5	15		10		3		1		1		1	

**Figure 3.**
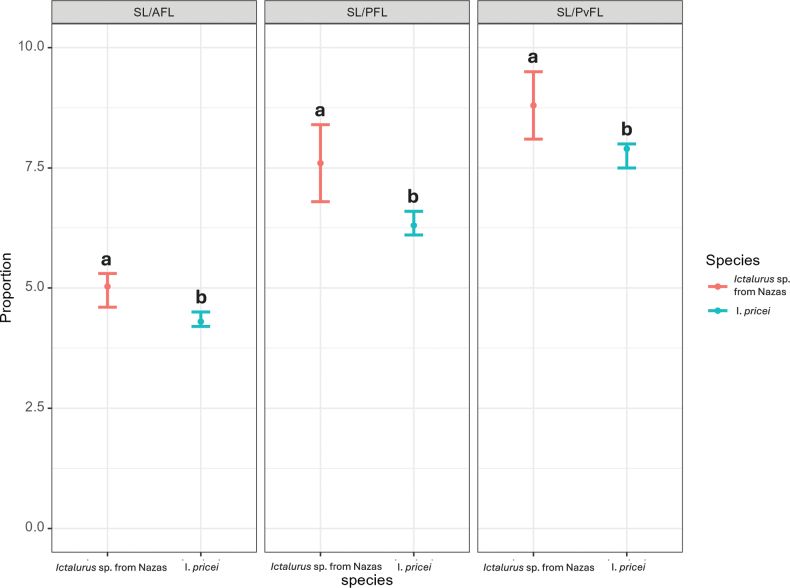
Three significant proportional characters distinguishing *Ictalurus* sp. from Nazas and *I.pricei*. Values are means with 95% confidence intervals. Different lowercase letters above the error bars indicate significant differences (*P* < 0.05).

In contrast to the meristic data, the PCA based on linear measurements did not indicate a clear differentiation pattern (Suppl. materials 1, 2: table S2, fig. S1). However, for the proportions of linear measurements, the Student’s t-test revealed significant differences in three proportions: SL/AFL (t = 3.4, p = 0.0009), SL/PFL (t = 3.8, p = 0.0003) and SL/PvFL (t = 3.1, p = 0.002). In all three cases, *Ictalurus* sp. from Nazas presented the lowest proportion values (Fig. 3).

**Figure 4.**
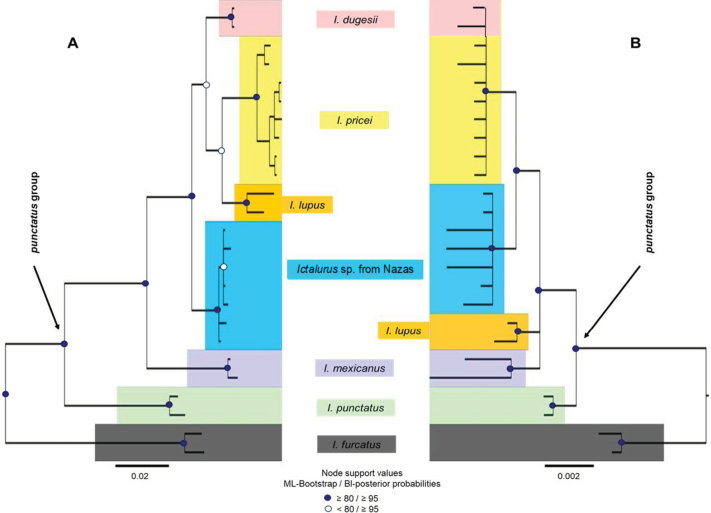
Bayesian inference and maximum likelihood hypothesis of the genus *Ictalurus*. Hypothesis based on A. Mitochondrial gene *Cytb*, and B. Nuclear gene *RAG1*

The phylogenetic analyses included sequences from 11 recognized taxa, comprising six recognized species of *Ictalurus*, including *Ictalurus* sp. from Nazas, and five outgroups. The final amplicon lengths were 1125 bp for *Cytb* and 1404 bp for *RAG1*. The HKY+F+G4 model was selected as the best fit for the *Cytb* dataset, while the K2P model was chosen for *RAG1*. Using Bayesian inference and maximum likelihood, the phylogenetic analyses revealed seven groups in the topology based on *Cytb* (Fig. 4A), recovering the six recognized species as monophyletic groups, plus *Ictalurus* sp. from Nazas. In contrast, the topology based on the nuclear locus revealed six groups, recovering only four recognized species as monophyletic groups, a further group formed by *I.pricei* and *I.dugesii*, and another comprising *Ictalurus* sp. from Nazas (Fig. 4B).

*P*-distances between the members of *Ictalurus* ranged from 2.1 to 9.9% for *Cytb* (Table 6) and from 0.1 to 1.6% for *RAG1* (Table 6). Based on the meristic, morphometric, and molecular evidence, a new species of the genus *Ictalurus* was described. Descriptive statistics of the meristic data, linear measurements, and proportions for the holotype and paratypes are presented in Tables 4, 5.

**Table 5.** Linear measurements of *Ictalurus* sp. from Nazas (abbreviations of proportions are shown in the Materials and method section). *N* = sample size, SD = standard deviation.

	Ictalurus sp. from Nazas				I.pricei		
	Holotype	Paratype *N* = 23			*N* = 36		
		Range	Mean	SD	Range	Mean	SD
Total length	342	106.4-388.6	193.3	98.7	102.9-290	179.3	46.3
Standard Length	302	87.9-347	169.3	89.8	87.1-208	154.2	38.9
Head length	70.2	23.6-81.8	41.6	18.8	22.3-53.9	39.2	9.9
HL/ID	2.3	2.2-3.2	2.6	0.3	2.1-3	2.6	0.2
HL/ED	8.2	4.6-9.6	6.6	1.4	4.1-7.2	5.9	0.7
SL/HL	4.3	3.4-5.1	4.0	0.4	3.6-4.2	3.9	0.1
SL/MH	5.4	4.2-9.4	5.6	1.4	3.8-6.1	4.7	0.6
SL/AFL	6.0	2.9-6.7	5.0	0.8	3.6-5.1	4.4	0.4
SL/PFL	8.7	5.8-11.4	7.6	1.8	4.4-7.6	6.4	0.7
SL/PvFL	10.7	6.7-12.5	8.8	1.6	6.8-8.9	7.9	0.5

**Table 6.** Uncorrected pairwise genetic distances (in percentages) among members of the *punctatus* group, based on mitochondrial and nuclear loci. Values above the diagonal represent distances based on *Cytb*, while values below the diagonal represent distances based on *RAG1*.

	I.dugesii	I.furcatus	I.lupus	I.mexicanus	I.nazas sp. nov.	I.pricei	I.punctatus
I.dugesii							
I.furcatus	9.4/1.6						
I.lupus	2.5/0.3	9.5/1.3					
I.mexicanus	4.7/0.3	10.7/1.6	5.5/0.4				
I.nazas sp. nov.	2.1/0.6	9.6/1.7	2.9/0.5	5.9/0.7			
I.pricei	2.5/0.1	9.9/1.6	2.6/0.3	5.4/0.3	3.0/0.6		
I.punctatus	7.3/0.5	9.3/1.1	8.2/0.3	7.6/0.5	7.3/0.7	7.6/0.5	

### ﻿ Ictalurus nazas sp. nov.

Taxon classificationAnimaliaSiluriformesIctaluridae

2512F819-1611-54AB-ACC1-953C9DE8D558

https://zoobank.org/A26FD24E-C28C-4C72-8D82-BD3995E470D2

**Figure 5.**
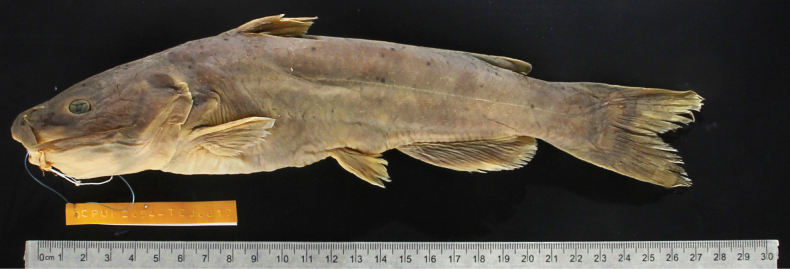
*Ictalurusnazas* sp. nov. holotype, UMSNH-2654, adult, SL 302 mm, Ramos River, 1.12 km north of El Olote, Santiago Papasquiaro, Durango, Mexico, endorheic drainage Ramos River.

Fig. 5 , Tables 4 , 5

#### Type locality.

Ramos River, 1.12 km north of El Olote, Santiago Papasquiaro, Durango, Mexico.

#### Type materials.

***Holotype*.** • UMSNH-2654, adult, 302 mm SL, Ramos River 1.12 km north of El Olote, Santiago Papasquiaro, Durango, Mexico, endorheic drainage Ramos River; 25°14'13.2"N, -105°27'0.4"W, collected on June 2, 2008. ***Paratypes*.** • UMSNH-7266 (*N* = 1), El Olote, Santiago Papasquiaro, Durango, Mexico, endorheic drainage Ramos River; 25°13'17.04"N, -105°26'37.79"W, collected on May 10, 2004.; • UMSNH-1642 (*N* = 3) river to the east of Santiago Papasquiaro, Durango, Mexico, endorheic drainage Ramos River; 25°2'26.12"N, -105°24'54.67"W, collected on September 19, 2005; • CNPE-IBUNAM-17795 (*N* = 5) Nazas River, main body, Nazas, Durango, Mexico, endorheic drainage Nazas River; 25°12'57"N, -104°10'25"W, collected on April 4, 2004; • CNPE-IBUNAM-17779 (*N* = 1) Dolores Hidalgo, Nazas, Durango, Mexico, endorheic drainage Nazas River; 25°15'59"N, -104°05'24"W, collected on April 4, 2004; • CP-UJED-0018 (*N* = 1) Balneario “Belem” Peñón Blanco, Durango, Mexico, endorheic drainage Nazas River; 24°44'36"N, -104°04'36"W, collected on September 18, 1989; • CPUJED-0001 (*N* = 4 El Ranchito, Peñón Blanco, Durango, Mexico, endorheic drainage Nazas River; 24°59'19"N, -104°19'39"W, collected on August 5, 2015; • CPUJED-0002 (*N* = 2) Arroyo Covadonga, Nazas, Durango, Mexico, endorheic drainage Nazas River, 25°11'31"N, -104°11'41"W, collected on December 13, 2022; • CPUJED-0003 (*N* = 1) Amoles, Nazas, Durango, Mexico, endorheic drainage Nazas River, 25°07'47"N, -104°29'47"W, collected on December 1, 2022; • CPUJED-0004 (*N* = 4) Nuevo Covadonga, Peñón Blanco, Durango, Mexico, endorheic drainage Nazas River, 24°54'35"N, -104°04'47"W; collected on May 22, 2023; • CPUJED-0005 (*N* = 1) El Ranchito, Peñón Blanco, Durango, Mexico, endorheic drainage Nazas River; 24°59'19"N, -104°19'39"W, collected on May 21, 2023.

#### Diagnosis.

**Figure 6.**
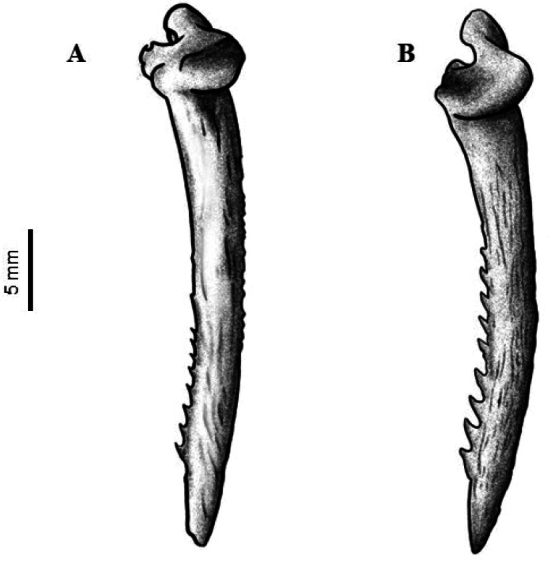
View of the pectoral spine of *Ictalurus* species: A. *I.nazas* sp. nov., 33 mm. and B. *I.pricei*, 28 mm. *Ictalurusnazas* sp. nov. exhibits fewer posterior dentations, which are underdeveloped and can only be distinguished from the middle of the spine towards the apex.

*Ictalurusnazas* sp. nov. differs from *I.pricei* by the following combination of characters: 16–18, rarely 19, anal fin rays (vs. 19–24); shorter premaxillary barbel, reaching only to the opercle margin (vs. extending beyond the margin of the opercle); shorter base of anal fin, 5.0 times into the SL (vs. larger base, 4.4 times into the SL); shorter length of pectoral fin, 7.6 times into the SL (vs. larger pectoral fin, 6.4 times into the SL); shorter length of pelvic fin, 8.8 times into the SL (vs. larger pelvic fin, 7.9 times into the SL); lower number of posterior dentations, 5–8, rarely 9 and 10, (vs. higher number of dentations, 8–11, rarely 12–14 dentations) (Fig. 6); weaker development of dentations (vs. stronger development) (Fig. 6), and dentations concentrated in the apical part of the pectoral spine (vs. dentations extended from apical to median part of the pectoral spine) (Fig. 6).

In relation to other recognized species of *Ictalurus* (sensu Pérez-Rodríguez et al. 2023), *Ictalurusnazas* sp. nov. can be distinguished from its congeners, *I.lupus* (Miller et al. 2005), *I.dugesii* (Arce-H. et al. 2016), *I.mexicanus* (Arce-H. et al. 2016), *I.punctatus* (Miller et al. 2005), *I.furcatus* (Miller et al. 2005), and *I.balsanus* (Mejía et al. 2013), by presenting an anal fin base shorter than the head length, with 16 to 19 anal fin rays. Additionally, the premaxillary barbels do not extend beyond the posterior margin of the opercle (see Description for further details). Adult specimens of *Ictalurusnazas* sp. nov. also differ from most congeners, except *I.dugesii*, by exhibiting fewer and less developed posterior dentations on the pectoral spine, which are restricted to its apical portion.

Moreover, *Ictalurusnazas* sp. nov. can be diagnosed by the following characters: from *I.punctatus* by having a moderately forked caudal fin with a distal margin forming an “U” shape and fully rounded (vs. deeply forked caudal fin with a “V” shaped margin and pointed tips); from *I.lupus*, by having 7 to 9 (rarely 6 and 10) pectoral fin rays (vs. 9 to 10 rays); from *I.mexicanus*, by the presence of posterior dentations in the pectoral spine (vs. absence); from *I.dugesii* by presenting a blunt tip on the pectoral spine (vs. sharp tip); from *I.furcatus* by presenting fewer anal fin rays (16 to 19 vs. 28–38 rays), and from *I.balsanus* by presenting 17 to 19 caudal fin rays (vs. 22 rays).

#### Description.

Elongated body; large head, ventrally flattened, tapering anteriorly. Eyes small, diameter, 9.6 times into head the length, interorbital distance, 3.2. times into the head length. Terminal mouth position with eight barbels: four maxillary, two premaxillary, and two nasals. The premaxillary barbels do not reach the edge of the operculum.

Dorsal fin with 5 to 7, rarely 9 rays, its origin positioned at the midpoint of the pectoral fins. Pectoral fin length 8.4 times into the SL and contains 7 to 9 rays, though, on occasions, specimens may exhibit 6 or 10 rays. The pectoral spine features a series of 5 to 8 posterior dentations with weaker development; in rare cases, 9 or 10 dentations may be present. Pelvic fin is short, 9.5 times into the SL, with 7 to 8 rays, rarely 6 and 9; origin posterior to the base of the dorsal fin. Anal fin base shorter than head length, its length fitting 5.0 times into the SL; fin composed of 16 to 19 rays. Caudal peduncle short and moderately forked, with a rounded distal margin forming a “U” shape, with 17 to 19 rays.

Color. In life, the body is olive green, with a slightly darker pigmentation on the dorsal part of the head, fins, and premaxillary barbels. Ventral region, along with the maxillary and nasal barbels, appears noticeably lighter. The body features a few dark spots, while the caudal fin margin is distinctly black. In alcohol, the body takes on a gray hue, with the fins and ventral region significantly lighter. The fin margins remain black, and the premaxillary barbels appear darker than the maxillary and nasal barbels.

#### Habitat and ecology.

The species inhabits slow-moving waters with temperatures ranging from 17 to 22 °C. It is generally found in areas with exposed bedrock slabs and crevices that provide refuge.

#### Distribution.

**Figure 7.**
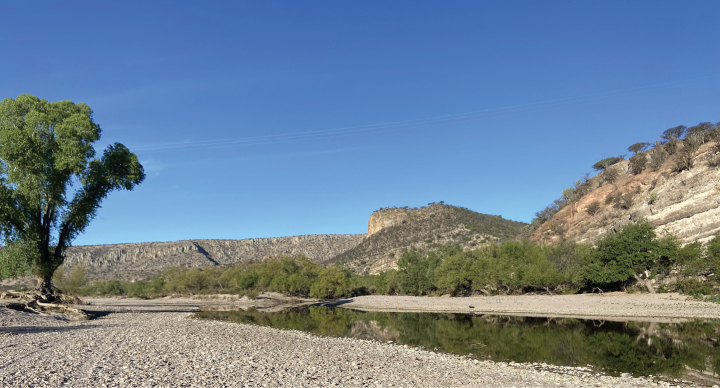
Type locality of *I.nazas* sp. nov. Ramos River, 1.12 km north of El Olote, Santiago Papasquiaro, Durango, Mexico, endorheic drainage, Ramos River; 25°14'13.2"N, -105°27'0.4"W.

*Ictalurusnazas* sp. nov. is only known from the endorheic basin of the Nazas-Aguanaval System, including its main tributaries: the Rivers Peñón Blanco and Ramos (Fig. 7).

#### Etymology.

The specific epithet nazas is derived from the name of the basin in which the species is distributed and to which it is endemic.

## ﻿Discussion

Although *Ictalurusnazas* sp. nov. was considered an undescribed species during the last century (sensu Miller 1986; Smith and Miller 1986), the studies that were conducted were limited to lists and regionalization of the ichthyofauna from biogeographical provinces of North America, without performing a detailed morphological comparison or description. The strength of the molecular evidence, combined with increased taxonomic sampling within *Ictalurus*, has provided more evidence for the recognition of this new taxon and further contributed to resolving the systematics of this genus. The diagnostic morphological characters of *I.nazas* sp. nov. are distinct and differentiate this new taxon, particularly from *I.pricei*, the nominal species to which it was originally assigned. These same characteristics, in combination with others, have also proved useful for clearly distinguishing this new taxon from the remaining species of *Ictalurus* (see Dichotomous key). Although the two phylogenetic trees showed minor inconsistencies with each other, both loci consistently recovered *I.nazas* sp. nov. within the *punctatus* group as a well-supported, reciprocally monophyletic lineage (Fig. 4). Uncorrected *p*-distances based on *Cytb* between *I.nazas* sp. nov. and its closest congeners ranged from 2.1% to 2.9% (Table 6), which falls within the minimum interspecies divergence limits for *Cytb* within Ictaluridae (1.8–3.6%) (Hardman and Page 2003; Hardman 2004; Egge and Simons 2006; Egge et al. 2015; Pérez-Rodríguez et al. 2023). Both the phylogenetic relationships and molecular divergence estimates indicate an independent evolutionary history that began approximately five million years ago (Janzen et al. 2023; Pérez-Rodríguez et al. 2023). This history is consistent with the isolation of *I.nazas* sp. nov. in the ancient Nazas River paleosystem during the Pliocene (Domínguez-Domínguez et al. 2011) and constitutes strong evidence to support its recognition as a new and independent taxonomic entity.

This finding is consistent with other endemic fish species from the Nazas River, such as the leuciscids *Notropisnazas* Meek, 1904 (Contreras-Balderas et al. 2005), *Gilaconspersa* Garman, 1881 (Contreras-Balderas et al. 2005; Schönhuth et al. 2014), *Cyprinellagarmani* Jordan, 1885 (Contreras-Balderas et al. 2005), and *Cyprinellaalvarezdelvillari* Contreras-Balderas & Lozano-Vilano, 1994 (Contreras-Balderas et al. 2005), the catostomid *Pantosteusnebuliferus* (Garman, 1881) (Contreras-Balderas et al. 2005; Corona-Santiago et al. 2018), and the cyprinodontid *Cyrpinodonnazas* Miller, 1976 (Echelle et al. 2005). These findings have important biogeographic and conservation implications. Although the Nazas River basin is currently an endorheic system, it has been considered a historical transitional zone between freshwater fish lineages originating from the Gulf of Mexico and Pacific slope drainages in North America. This is supported by evidence of the colonization of the genus *Moxostoma* Rafinesque, 1820b from the Gulf slope into the Pacific slope drainages during the early Pliocene (Pérez-Rodríguez et al. 2016). The two phylogenetic hypotheses, based on *Cytb* and *RAG1* presented herein, suggest that the ancestor of *Ictalurusnazas* sp. nov. and the species currently distributed in the Pacific slope drainages (*I.pricei* and *I.dugesii*) likely inhabited the region corresponding to the paleo-Nazas system. Thus, this area also appears to have served as a transitional zone between *Ictalurus* species from the Gulf of Mexico slope and those from the Pacific slope drainages. Indeed, previous phylogenetic hypotheses and divergence time estimates (Janzen et al. 2023; Pérez-Rodríguez et al. 2023) indicate that the ancestor of *Ictalurusnazas* sp. nov. originated in the early Pliocene, supporting a biogeographic transition similar to that hypothesized for *Moxostoma* species. The addition of this new taxon increases both the richness of the genus *Ictalurus* in Mexico and the level of endemism for the Nazas River, suggesting that the Nazas River basin should be considered an important biogeographic province in North America that merits additional conservation protections.

We further highlight the fact that this study is part of an extensive investigation throughout the Nazas basin, with a high completeness of sampling within the basin and its tributaries. Despite this, only specific populations of *I.nazas* sp. nov. were found at the sites mentioned above. Immediate management and conservation measures are therefore recommended, together with further research on the biology and ecology of this species. These studies could ultimately support the inclusion of *I.nazas* sp. nov. in vulnerability categories at both national and international levels, ensuring that conservation efforts are appropriately directed, given its status as a microendemic species.

### ﻿Dichotomous key to the *Ictalurus* species of the *punctatus* group

**Table:** 

1	Caudal fin deeply forked, with its distal margin in a U-shape and pointed tips; 26 to 32 anal fin rays	***Ictaluruspunctatus***
–	Caudal fin moderately forked, with its distal margin in a V-shape and semi-rounded to rounded tips; 27 or fewer anal fin rays	**2**
2	Pectoral spine with numerous strong dentations along its posterior margin; head length shorter than anal fin base length	**3**
–	Pectoral spine smooth or corrugated, with few weak posterior dentations; head length equal to or greater than anal fin base length	**4**
3	Insertion of the dorsal fin begins at the midpoint of the pectoral fin; 9 to 10 pectoral fin rays; 23 to 25 anal fin rays	***Ictaluruslupus***
–	Insertion of the dorsal fin begins at the posterior tip of the pectoral fin; 11 pectoral fin rays; 18 to 24 anal fin rays	***Ictaluruspricei***
4	18 to 24 anal fin rays; maxillary barbels extend to the insertion of the pectoral fin or beyond pectoral spine with few or no weak dentations; the tip of the pectoral spine is sharp; the distal margin of the caudal fin is semi-rounded	**5**
–	16 to 19 anal fin rays; maxillary barbels do not extend to the insertion of the pectoral fin; pectoral spine with few weak dentations visible from the middle to the tip; tip of the pectoral spine is blunt; distal margin of the caudal fin is completely rounded	***Ictalurusnazas* sp. nov.**
5	20 to 24 anal fin rays; pectoral spine with few weak dentations visible from the middle to the tip	***Ictalurusdugesii***
–	20 to 21 anal fin rays; posterior face of the pectoral spine lacks dentations	***Ictalurusmexicanus***

### ﻿Comparative material

*Ictaluruspricei*: CNPE-IBUNAM-5712 (*N* = 2) Aros River, to the south of Natora, Son., Sahuipa, Sonora, Mexico; UMSNH-2931, (*N* = 1), Bavispe River, at 8.85 km east of Col. Morelos, Bavispe, Sonora, Mexico; UMSNH-7763, (*N* = 3), Moctezuma River, to the south of Caserio de Terapa, Moctezuma, Sonora, Mexico; UMSNH-2221, (*N* = 5), Bavispe River, at 7.2 km northeast of the village de Huachinera, Bacerac, Sonora, Mexico; UMSNH-2642, (*N* = 5), Guerachi River, at 27 km southeast of Guachochi city, Guadalupe y Calvo, Chihuahua, Mexico; UMSNH-2534 (*N* = 5), Guerachi River, to southeast of Guachochi city, Guadalupe y Calvo, Chihuahua, Mexico; UMSNH-7764 (*N* = 6), Guerachi River, at 27 km southeast of Guachochi city, Guadalupe y Calvo, Chihuahua, Mexico; CPUJED-0008 (*N* = 10), El Sauzal, Urique, Chihuahua, Mexico; CNPE-IBUNAM-20776 (*N* = 2) San Isidro Stream, Rosario, Sinaloa, Mexico.

## Supplementary Material

XML Treatment for Ictalurus nazas

## ﻿Additional information

### Use of AI

No use of AI was reported.

### Author ORCIDs

Edith Avila-Treviño https://orcid.org/0009-0002-4728-6942

Gabriel Fernando Cardoza-Martínez https://orcid.org/0000-0002-6328-6558

Fernando Alonzo-Rojo https://orcid.org/0000-0001-8529-6100

Rodolfo Pérez-Rodríguez https://orcid.org/0000-0002-0822-4610

### Data availability

All of the data that support the findings of this study are available in the main text or Supplementary Information.

## Supplementary materials

Supplementary material 1 Supplementary information Data type docx Explanation note **table S1.** Voucher specimens, gene, and GenBank accession number of analyzed sequences of the species used as the outgroup. **table S2.** Principal Component Loadings (PC Loadings) of meristic characters used for the discrimination of *Ictalurus* sp. from Nazas (*N* = 24) and *I.pricei* (*N* = 36). https://binary.pensoft.net/file/1387561This dataset is made available under the Open Database License (http://opendatacommons.org/licenses/odbl/1.0/). The Open Database License (ODbL) is a license agreement intended to allow users to freely share, modify, and use this Dataset while maintaining this same freedom for others, provided that the original source and author(s) are credited.Edith Avila-Treviño, Gabriel Fernando Cardoza-Martínez, Fernando Alonzo-Rojo, Rodolfo Pérez-Rodríguez

Supplementary material 2 Principal component analysis for linear characters used for species discrimination between *Ictalurus* sp. from Nazas (*N* = 24) and *I.pricei* (*N* = 36) Data type docx https://binary.pensoft.net/file/1387562This dataset is made available under the Open Database License (http://opendatacommons.org/licenses/odbl/1.0/). The Open Database License (ODbL) is a license agreement intended to allow users to freely share, modify, and use this Dataset while maintaining this same freedom for others, provided that the original source and author(s) are credited.Edith Avila-Treviño, Gabriel Fernando Cardoza-Martínez, Fernando Alonzo-Rojo, Rodolfo Pérez-Rodríguez
