# Supplementary material for: ﻿Ictalurusnazas sp. nov., a new species of North American catfish (Siluriformes, Ictaluridae) from Mexico
Source: Zookeys. 2025 Aug 4;1248:33–51. doi: 10.3897/zookeys.1248.151641 (PMC12340532; doi:10.3897/zookeys.1248.151641)
Supplement: Supplementary material 1 — Supplementary information [file zookeys-1248-033_article-151641__-s001.docx]

**Supplementary material 1**

**Table S1.** Voucher specimens, gene, and GenBank accession number of analyzed sequences of the species used as the outgroup.

| **Taxa** | **Locality** | **Voucher tissue** | ***Cytb*** | ***RAG1*** |
| --- | --- | --- | --- | --- |
| *Bagrus ubangensis* Boulenger, 1902 | Democratic Republic of the Congo: Lualaba | AUM 51278 | OQ559148 | OQ567208 |
| *Cranoglanis henrici* (Vaillant, 1893) | Red River basin, Ha Moi, Vietnam | ANSP 203039 | OQ559153 | OQ567213 |
| *Pylodictis olivaris* (Rafinesque, 1818) | Mexico: San Luis Potosi, Panuco | UMSNH 57621 | OQ559224 | OQ567288 |
| *Ameiurus melas* (Rafinesque, 1820) | Claireville Reservoir, Ontario, Canada | ROM 100161 | OQ559136 | OQ567194 |
| *Noturus gladiator* Thomas & Burr, 2004 | Hatchie River, Mississippi, USA | MMNS 67765 | OQ559195 | OQ567256 |

**Table S2.** Principal Component Loadings (PC Loadings) of meristic characters used for the discrimination of *Ictalurus* sp. from Nazas (*N* = 24) and *I. pricei* (*N* = 36).

|  | PC1 | PC2 | PC3 | PC4 | PC5 | PC6 | PC7 | PC8 | PC9 | PC10 | PC11 | PC12 |
| --- | --- | --- | --- | --- | --- | --- | --- | --- | --- | --- | --- | --- |
| Standard length (SL) | 0.927 | -0.299 | -0.018 | -0.046 | -0.016 | 0.184 | 0.098 | 0.050 | -0.040 | 0.012 | -0.010 | -0.007 |
| Head length (HL) | 0.201 | 0.105 | -0.014 | 0.317 | 0.112 | -0.389 | -0.345 | -0.549 | 0.265 | 0.131 | -0.410 | 0.092 |
| Caudal peduncle length (CPL) | 0.115 | 0.217 | 0.127 | -0.648 | 0.545 | -0.400 | 0.155 | 0.063 | 0.131 | -0.006 | -0.002 | -0.026 |
| Maximum height (maxH) | 0.117 | 0.646 | -0.354 | 0.304 | 0.271 | 0.305 | 0.423 | -0.032 | -0.011 | -0.008 | -0.065 | 0.011 |
| Minimum height (minH) | 0.057 | 0.098 | 0.018 | 0.007 | 0.097 | 0.035 | -0.148 | -0.346 | -0.114 | 0.528 | 0.737 | 0.013 |
| Eye diameter (ED) | 0.017 | 0.059 | -0.020 | -0.006 | -0.002 | 0.018 | -0.114 | -0.055 | -0.091 | 0.051 | -0.091 | -0.980 |
| Interorbital distance (IO) | 0.094 | 0.052 | -0.025 | 0.165 | -0.095 | -0.176 | 0.019 | -0.076 | 0.497 | -0.630 | 0.505 | -0.123 |
| Anal fin base length (AFL) | 0.141 | 0.382 | -0.319 | -0.328 | -0.717 | -0.302 | 0.023 | 0.028 | -0.035 | 0.122 | -0.017 | 0.037 |
| Pectoral fin length (PFL) | 0.073 | 0.295 | -0.020 | -0.161 | 0.064 | 0.417 | -0.685 | 0.339 | 0.336 | 0.039 | -0.025 | 0.062 |
| Pelvic fin length (PvFL) | 0.075 | 0.172 | -0.037 | -0.120 | 0.093 | 0.026 | -0.348 | -0.257 | -0.680 | -0.528 | 0.041 | 0.100 |
| Body width (BW) | 0.123 | 0.390 | 0.865 | 0.137 | -0.219 | 0.032 | 0.110 | 0.034 | -0.061 | 0.003 | -0.030 | 0.002 |
| Lower jaw length (LJL) | 0.120 | 0.066 | -0.074 | 0.438 | 0.150 | -0.512 | -0.175 | 0.616 | -0.250 | 0.093 | 0.133 | -0.003 |
