# Supplementary material for: ﻿Ictalurusnazas sp. nov., a new species of North American catfish (Siluriformes, Ictaluridae) from Mexico
Source: Zookeys. 2025 Aug 4;1248:33–51. doi: 10.3897/zookeys.1248.151641 (PMC12340532; doi:10.3897/zookeys.1248.151641)
Supplement: Supplementary material 2 — Principal component analysis for linear characters used for species discrimination between Ictalurus sp. from Nazas (N = 24) and I.pricei (N = 36) [file zookeys-1248-033_article-151641__-s002.docx]

**Supplementary material 2**


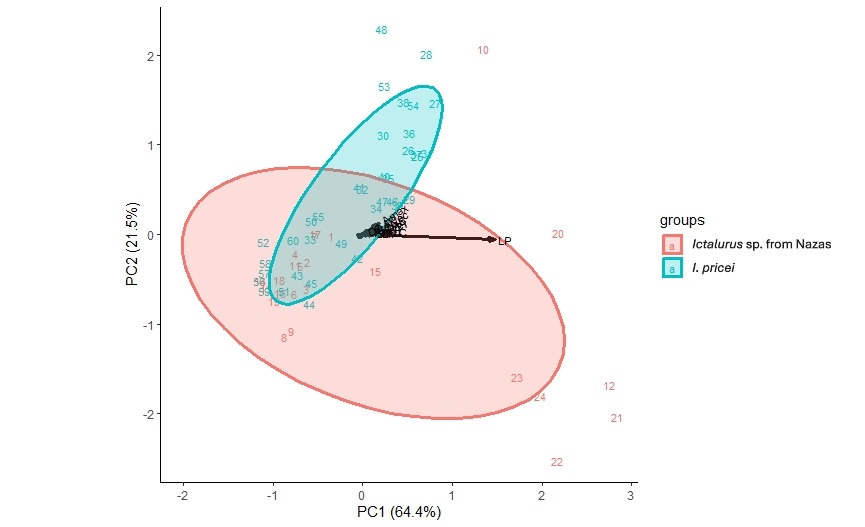


**Figure S1.** Principal Component Analysis for linear characters used for species discrimination between *Ictalurus* sp. from Nazas (*N* = 24) and *I. pricei* (*N* = 36).
